# Prevalence of hip femoroacetabular impingement deformities in high-level (La Liga) male professional football players

**DOI:** 10.1186/s12891-024-07247-x

**Published:** 2024-02-21

**Authors:** Rafael Arriaza, Miguel Saavedra-García, Alvaro Arriaza, Antonio Cruz-Cámara, Manuel Leyes, Luis Cerezal, Antonio Maestro

**Affiliations:** 1 Instituto Médico Arriaza y Asociados, A Coruña, Spain; 2 Grupo INCIDE-Universidade da Coruña, A Coruña, Spain; 3 Centro Traumatólogos Santander, Cantabria, Spain; 4 Centro Olympia, Madrid, Spain; 5 Centro Diagnóstico Médico Cantabria, Santander, Spain; 6 Hospital Begona, Gijón, Spain

**Keywords:** Femoroacetabular impingement, Professional football players, Footballer’s hip, Hip deformity

## Abstract

**Background:**

Femoroacetabular impingement (FAI) is considered one of the main causes of hip osteoarthritis in young adults, especially in athletes. In recent years, morphological changes in FAI in the hip have been linked to early and intense sports participation, but studying top-level athlete samples is not easy. This paper presents the prevalence of FAI radiological markers in 120 active white male professional football players in the Spanish First Division League (La Liga) and compares the morphological changes with those of a control group of healthy individuals without significant sport activity.

**Methods:**

The precontract medical evaluation hip X-rays of 120 white male professional football players from four different First Division Spanish football teams were prospectively filed and retrospectively reviewed by a dedicated skeletal radiologist. The footballers’ hip X-rays were compared with those of a control group of 80 healthy individuals (age-sex matched) without significant sport activity (obtained from routine work medical checks).

**Results:**

The femoral head-neck deformity associated with the Cam type of femoroacetabular impingement was observed in 61.6% of professional football players and only in 11.6% of the control group (*p* <0.01). The presence of “herniation pit” (11.6%) and os acetabuli (13.3%) also reached statistical significance in the professional football players group. In the other analyzed parameters, no statistically significant differences between the groups were observed.

**Conclusions:**

White professional top-level football players have an increased incidence of abnormal lateral epiphyseal extension ("pistol grip deformity"), os acetabuli and herniation pits.

## Introduction

Femoroacetabular impingement (FAI) has become a well-recognized clinical concept and is believed to increase the risk for early-onset osteoarthritis (OA), especially in highly active patients [1]. Although it is currently well recognized that pure radiologically asymptomatic abnormal morphology should be differentiated from FAI syndrome, which is a motion-related clinical disorder of the hip with a triad of symptoms, clinical signs and imaging findings [2], it represents premature contact between the proximal femur and the acetabulum that can become symptomatic or lead to early-onset hip osteoarthritis, especially in persons who submit the hip joint to repetitive, high-amplitude forced movements [3].

The prevalence of FAI as a radiological diagnosis is estimated to be 10%–15% in a general adult population [4, 5], but its prevalence in different “at risk” populations is still under study [6], although some studies have shown a greater prevalence in professional or top-level football players [7]. Development of hip impingement seems to occur most frequently in sports involving quick accelerating weight-bearing motions and sudden changes in direction, such as football, tennis, and handball, and radiographic indicators of FAI are very common among athletes evaluated at the National Football League Scouting that were subjected to radiographic examination for the clinical suspicion of hip disease [8]. Standard radiographic examination must include as usual protocol in football players the assessment of the femoral head-neck junction, the shape of the femoral head and acetabular roof, and the contour and shape of the acetabular rim [9], but assessment of acetabular depth, inclination, and version is also important. Fibrocystic changes (FCCs) in the epiphyseal vicinity should also be noted, as there is growing evidence that these radiolucencies, first described in 1982 as herniation pits [10], may develop secondary to the impingement process [11].

The development of symptoms in FAI results from abnormal contact between the proximal femur (head, neck and head-neck junction) and the acetabular rim, which causes labrum and cartilage tears. It is classified as either the cam or pincer type on the basis of the underlying anatomic deformity. Patients complain of groin pain that occurs during athletic activities or prolonged sitting. Episodes of locking may occur, mainly related to unstable labral tears. At physical examination, the impingement test reveals a decreased range of motion and painful limitations during flexion, especially when both internal rotation and adduction of the hip are performed at the same time. Similar to the sensitivities and specificities of these findings for impingement syndromes in other joints, the sensitivities and specificities of these clinical observations for FAI remain unknown [12].

Despite the frequent incidence of hip pathology in retired football players and the fact that football is the most popular sport in the world, the underlying frequency of FAI in professional players in highly competitive European leagues is not known [13].

The purpose of this study was to compare in a randomized manner the prevalence of hip dysplasia (HD) and FAI radiological markers in 120 active white male professional football players in the Spanish First Division League compared to a control group of healthy white male individuals without significant sport activity during adolescence or juvenile age. We hypothesized that radiological abnormal hip morphology would be more common in the group of professional football players.

## Materials and methods

Before a professional contract is signed between a footballer and a club, a medical evaluation (which includes orthopedic and cardiological exams) is mandatory in Spain. The precontract medical evaluation hip X-rays of 120 white professional football players from four different First Division Spanish football teams (Atlético de Madrid, Racing de Santander, Real Sporting de Gijón, and Real Club Deportivo de La Coruña) were prospectively filed over 4 years and retrospectively reviewed by two dedicated skeletal radiologists. The authors were team doctors for those teams, and thus had access to the players’ images and files, and were responsible for the pre-contract medical evaluation and clearance. Inclusion criteria were the availability of an anteroposterior (AP) and a true lateral pelvic radiograph, both taken with a standardized technique, being active white professional footballers, and not being discarded due to medical reasons related to hip disorders or having undergone surgery for hip injuries. All football players were prospectively screened for (among others) hip and pelvis injuries before contract signature, and AP pelvis and lateral lumbar spine and pelvis weight-bearing radiographs were routinely obtained. For the AP view, hips were kept in a neutral abduction-adduction position, with toes directed forward, and the beam was directed to a point midway between the pubic symphysis and the line joining both anterior superior iliac spines [9]. The footballers’ hip X-rays were compared with those of a control group of 80 white healthy individuals (age-sex matched) without significant sport activity during adolescence or juvenile age (obtained from routine work medical checks). The control group sample was choosen matching for age and sex, and discarding those who had been involved in avid sporting activity during childhood or adolescence (i.e.: the age where it is supposed that hip deformity arises in response to the high mechanical demands of the sport). Z-test and T-test sample size calculator to detect Cohen’s medium effect size was performed with a significance level (α) set at 0.05 and power (1-β) set at 0.8 results into a sample size of 64 control males. We increased sample to 80 to reach a 0.882 power. Then with this sample size we can detect adequately the differences between the two samples. To detect small between-group effects, we would need a sample of about 400 subjects, much larger than the number of accessible soccer players in our sample. For this reason, we have settled for far more than 64 controls (up to 80), in order to increase the power of the study significantly. Patient identification was removed from all radiographs for patient confidentiality, and radiographs were independently assessed by two senior musculoskeletal-dedicated radiologists. Radiologists were blinded to which group the subjects belonged. To assess intraobserver variability, all plain films were reevaluated by one of the observers one month after the first assessment. Radiographic measurements were performed with free specific software (Hip2Norm) developed for the radiographic analysis of a digital AP pelvic radiograph for FAI [14].

Measurements obtained from AP pelvic film included: center-edge angle of Wiberg or LCE angle (angle formed by a line parallel to the longitudinal pelvic axis and by the line connecting the center of the femoral head with the lateral edge of the acetabulum), acetabular index or Tönnis angle (angle formed by a horizontal line and a tangent from the lowest point of the sclerotic zone of the acetabular roof to the lateral edge of the acetabulum), extrusion index (percentage of uncovered femoral head in comparison to the total horizontal head diameter), rate of acetabular coverage, and Shenton's line. Radiographic findings commonly associated with HD and FAI were recorded: herniation pit, os acetabuli, decreased antero-superior femoral head–neck offset (defined as a distance inferior to 8 mm), acetabular retroversion (defined as positive crossover sign), lateral acetabular overcoverage (CE angle > 40°) or coxa profunda (defined as medial acetabular border overlapping the ilioischial line).

Statistical analyses were performed, including mean, standard deviation, and confidence intervals of radiographic indices. The results were compared between groups (male footballers vs. male controls) with the use of the χ^2^ test for qualitative variables and 2-sample t test for the measurements of angles. Interobserver and intraobserver agreement were calculated using Cohen’s K statistic. The K values were rated in accordance with the rating system of Landis and Koch as follows: K=0–0.2, slight agreement; K=0.21–0.40, fair agreement; K=0.41–0.60, moderate agreement; K=0.61–0.80, substantial agreement; K=0.81–0.99, excellent agreement; and K=1.0, absolute agreement [15]. Statistical significance was defined as a *p*-value of <0.05. All tests were run using STATA statistical software 12/SE (Stata Corp, College Station, TX, USA).

The project was approved by the Research Ethics Committee of the University of A Coruña (CE 4/2017).

## Results

**Fig. 1:**
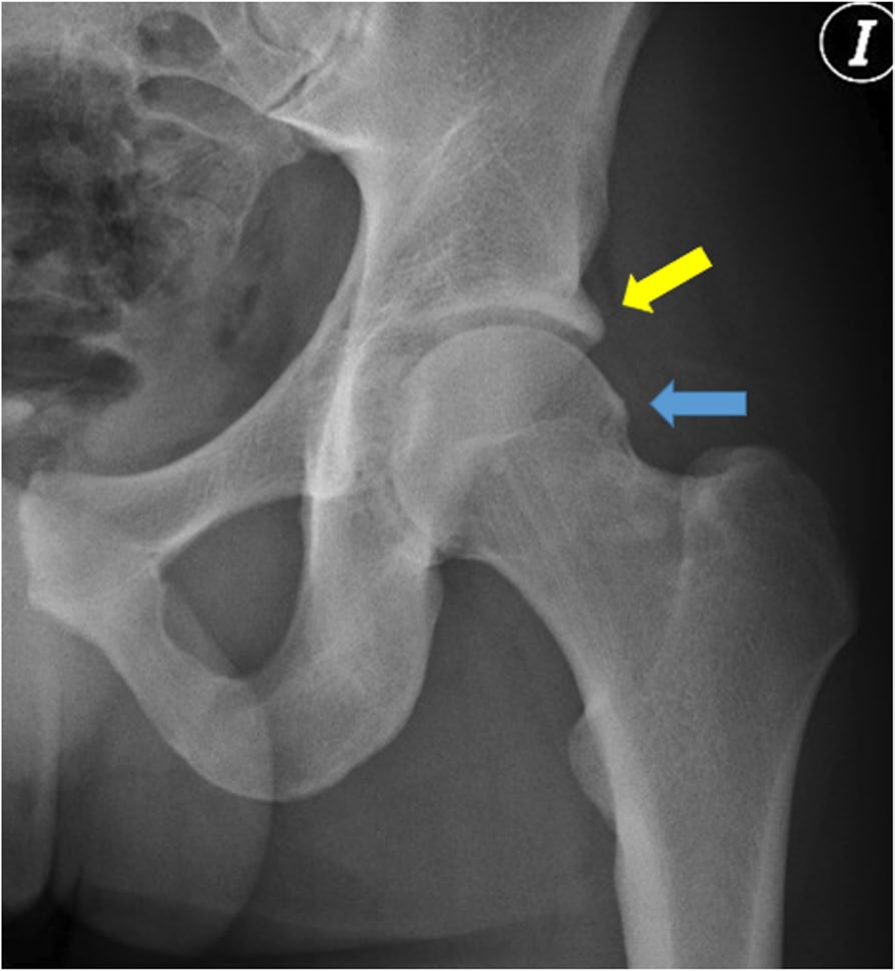
CAM type deformity (Blue arrow): prominence in the anterosuperior aspect of the proximal femur at the level of the head-neck junction) in the left hip of a 24 year old footballer accompanied by a pincer deformity (overcoverage) of the margin of the acetabulum (yellow arrow)

**Fig. 2:**
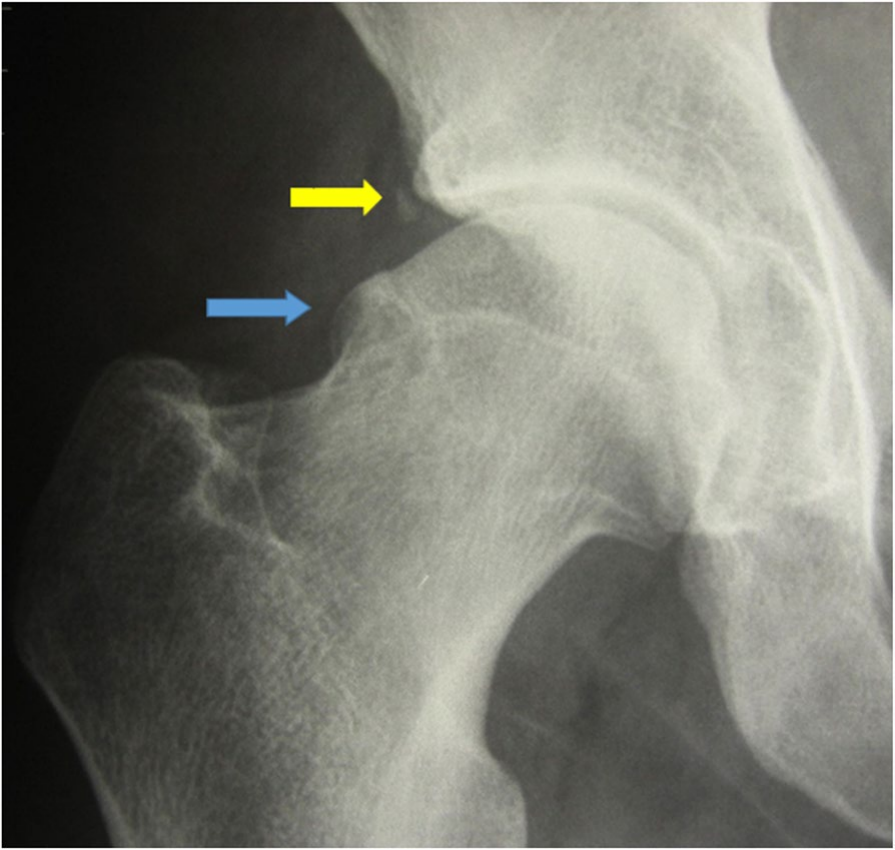
CAM type deformity (Blue arrow): prominence in the anterosuperior aspect of the proximal femur at the level of the head-neck junction in the right hip of a 31 year old footballer accompanied by a small os acetabuli at the margin of the acetabulum (Yellow arrow). In this case, the CAM prominence causes a loss of sphericity of the femoral head and a ‘bumpy‘ head neck-junction

The results of the analysis of the pelvis X-rays of the professional football players group and the control group are shown in Table 1. The femoral head-neck deformity associated with the cam type of FAI was observed in 61.6% of professional football players (Fig. 1) and only in 11.6% of the control group, reaching statistical significance (*p* <0.01). The presence of “herniation pit” (11.6%) and os acetabuli (Fig. 2) (13.3%) also reached statistical significance in the professional football players group. In all the other parameters analyzed (i.e., lateral center edge angle; acetabular index; extrusion index; crossover sign; posterior wall sign; depth of acetabular fossa; and ischial spine sign), no statistically significant differences between the groups were observed. Both the interobserver and intraobserver agreement were excellent in the diagnosis of these deformities, with an average value of 0.9 (Table 2).

**Table 1:** Radiological parameters analyzed in the study, showing the differences between the professional football players group and the control group, with the levels of statistical significance

	Professional Soccer Players	Control Group	*P* value
Lateral center edge angle (degrees)	34.8±3.87	33.4±3.45	0.44
Acetabular index (degrees)	5.7±0.8	6.1±1.2	0.73
Extrusion index (%)	17	16	0.84
Cross over sign Number and %	22 (18.3%)	14 (17.5%)	0.53
Posterior wall sign Number and %	18 (15%)	12 (15%)	0.36
Depth of acetabular fossa Number and %	28 (23.3%)	18(22.5%)	0.76
Ischial spine sign Number and %	24 (20%)	17 (21.2%)	0.23
Femoral head-neck deformity Number and %	74 (61.6%)	14(11.6%)	<0.01
Herniation pit Number and %	14(11.6%)	1 (1.2%)	<0.01
Os acetabuli Number and %	16(13.3%)	2(2.5%)	<0.01

**Table 2:** Interobserver reliability as related to the different radiologic parameters analyzed in the footballers’ hips

Observer 1 vs. Observer 2	Inter-rater reliability	95% Confidence interval	
		Minimum Level	Maximum Level
Lateral center edge angle	0.91	0.90	0.92
Acetabular index	0.89	0.87	0.91
Extrusion index	0.85	0.83	0.88
Cross over sign	0.85	0.82	0.87
Posterior wall sign	0.86	0.84	0.88
Depth of acetabular fossa	0.93	0.90	0.98
Ischial spine sign	0.92	0.89	0.94
Femoral head-neck defomity	0.89	0.87	0.92
Herniation pit	0.97	0.96	0.98
Os acetabuli	0.90	0.86	0.93

## Discussion

The main finding of this study is that the prevalence of hip FAI deformities affecting the femur in top-level white professional football players in the Spanish First Division League is higher than that in an age-matched control group of white male nonathletes. It is not easy that one single doctor can retrieve 120 professional football players’ hip X-rays in a highly competitive league to evaluate the hip morphology and the FAI markers because this is a very special population, but most likely, the findings of this paper will help both the physicians who act as consultants for professional football teams and those involved in the care of young footballers who try to reach professionalism to establish prophylactic protocols for these hips in risk patients. Maybe one of the missions of the sports doctors would be to counsel those young players who present with early clinical signs of FAI -and those who only have radiological markers of the problem without symptoms- on the importance of prevention and correct injury repair, or even on the convenience of pursuing a sport career in football if the chances of reaching success are unclear, given that only a small percentage of the adolescents engaged in football competition will finally make their career to professionalism [16]. Lohkamp et al. published a systematic review of OA and joint replacements of the lower limb and spine in ex-professional soccer players and found that the prevalence of hip OA and hip replacements is significantly higher in former players compared with the control group and considered that FAI could be a predisposing factor [17].

Altered morphology in the X-ray studies does not represent by itself a reason to discard a player from signing a contract, as our series show that 2/3 of the signed and active professional high-level players have radiological markers of FAI. This finding confirms those from Gerdhart et al. [7] in a sample of 75 male football players in the USA; Falotico et al. in Brazilian professional football players [6]; and those found in other high-level athletes in sports that require ample and high-velocity hip movements, such as ice-hockey [18], capoeira players [19], and American football [8]. It must be taken into account that the players in our study were all active white professionals who played in the Spanish Football First Division, which is considered by the UEFA as one of the most competitive leagues in Europe; this fact means that to reach a contract in this category, the players must have a very high level of skills and dedication and that most (if not all) of them started playing and entered competition in their early adolescence.

The fact that the deformities found in the radiological studies affect the femur and the acetabulum in different proportions probably implies that those deformities arise as a biological bone response to a mechanical stimulus during the growth period, as has been suggested by other authors [20]. These results suggest that FAI (especially cam type) can be related to the intensity of football practice during early adolescence [20], although no studies have tried to discover in how many of the football players who abandon either professionalism or their way into it, hip problems related to FAI were the cause of the drop off. There are very few prospective studies in asymptomatic professional high-level football players [7], and the study published in 2014 comparing semiprofessional and amateur football players had a sample of only 22 players in each group, although it was enough as calculated in the power analysis of the study [21]; in this study, the authors found that, in the semiprofessional group, 15 (62.5%) of 24 kicking legs (2 of the players kicked with two feet) had an increased α angle >55°, while 5 (27.3%) kicking legs of the amateur group had an α angle >55°. Even while footballers have a leg dominance, the number of drills and kicks that any professional performs with each leg made us decide not to analyze the results as related to the dominance.

If biased, the results found in our study would be so toward underestimation because only one AP view was used for the assessment of hip morphology alterations, with the possibility of missing anterolateral deformities. Due to the high number of X.-ray studies needed during the professional career of the players (the whole set of X-rays are repeated when they move to another team), team doctors usually try to reduce the number of radiological projections to a minimum. The prospective, highly selected population-based design strengthens the findings in our study. The standardized imaging protocol was also used.

Siebenrock et al. [22] studied a group of 37 male basketball players with a mean age of 17.6 years and compared them with 38 age-matched volunteers who had not participated in sporting activities at a high level and found an alpha angle of 55° or greater in 41 (89%) of 46 hips in the anterosuperior head quadrant, which represents a nearly 10-fold increase in the athletes' hips. A systematic revision from Mascarenhas et al. in 2016 showed that imaging suspicion of FAI is common among athletes, asymptomatic, and symptomatic populations, but significant differences in type and imaging signs of FAI exist among these groups that need to be considered in patients' decision making [23]. It is well known that male athletes, especially those practicing football, handball, and running and jumping sports, have an earlier onset and increased risk of osteoarthritis of the hip [13, 24]. The prevalence of hip osteoarthritis is 3 to 8.5 times higher in male athletes than in nonathletes, depending on the intensity of athletic activities and the physical loading of the hip [25]. Some studies have found an incidence of hip osteoarthritis of approximately 60% in retired high-level handball players, as opposed to a 10% incidence in a group of matched controls [26], and the systematic review from Petrillo et al. concluded that the prevalence rate of OA of both the hip and knee in male former professional football players was higher than that of age- and sex-matched individuals at a mean age of 55 years [27]. The prevalence rate of clinical OA of the hip in the male former professional football players was 8.6% (vs 5.6% of controls), while it was 21.2% for radiographic features of OA of the hip (vs 9.8% of controls). This finding may be explained by the effect of submitting a hip with a predisposing morphology to a hip-risk activity intensively. This fact could open the discussion about the role of the sports physicians, trainers and physiotherapist in counseling the young athletes with a physical examination or X-ray studies suggesting an anatomical hip alteration in regard to the choice of sports with less chances of harming those predisposed hips, especially taking into consideration that only a small percentage of those who enter the sports career aiming to professionalism will reach their goal [16] and may end with a premature hip osteoarthritis. Also, the high prevalence of radiological alterations in the pre-contract X-Rays of asymptomatic players should be known by the physicians in charge of the footballers’ evaluation to avoid misinterpreting the results and unccecessarily considering as pathological some frequent findings, while at the same time warning the players that in case hip related symptoms should arise, it is important to seek for medical advice as surgical remodelation might delay the necessity of a hip arthroplasty.

We must acknowledge some limitations to our study. First, only one AP radiographic view was available, without axial or Dunn projections. We are aware that several protocols have been suggested for the radiographic assessment of impingement, of which a supine AP and a cross-table lateral view seem to be preferred over others [9]. The supine AP view has traditionally been obtained with internally rotated hips, as the femoral necks project better in this position; thus, fractures are more easily detected. For the assessment of the acetabulum, however, a weight-bearing view in the anatomic position appears to be more appropriate as acetabular version is more correctly visualized. Furthermore, weight-bearing images are preferred for the measurements of joint space width [28]. It is reasonable to believe that two-dimensional imaging, as performed in our study, yields an underestimation of the prevalence of features suggestive of FAI, but the use of Hip2Norm software correction has shown an extremely good correlation with the findings of CT scans for the diagnosis of FAI radiological findings [14]. Additionally, the fact that only white footballers were selected in the sample could lead to an overestimation of the deformities in the whole group of professional footballers, as in professional football, there are usually players from different etnithities in a team, who may have different prevalences of hip deformities [29].

Maybe the field position and the number of kicks or dominance should be taken into account for future studies.

## Conclusions

In this study of white male professional players in the Spanish First Division Football League, there was a high prevalence of abnormal lateral epiphyseal extension (" pistol grip deformity"), as well as os acetabuli and herniation pit, compared to controls, which, at least until the time of the study, did not impede their participation in top-level football.

## Data Availability

The datasets analyzed during the current study are available from the corresponding author upon reasonable request.
